# Case Report: A Patient Diagnosed With Miller Fisher Syndrome and Myasthenia Gravis at the Same Time

**DOI:** 10.3389/fneur.2021.814453

**Published:** 2022-02-07

**Authors:** Nan Chen, Hanyu Cai, Jianhua Cheng

**Affiliations:** Department of Neurology, The First Affiliated Hospital of Wenzhou Medical University, Wenzhou, China

**Keywords:** Miller Fisher syndrome, myasthenia gravis, GQ1b, GT1a, titin

## Abstract

In this case report, we describe a patient who was first diagnosed with Miller Fisher syndrome (MFS) combined with myasthenia gravis (MG). A 58-year-old male patient presented with acute dysarthria with dizziness, ophthalmoplegia, absence of deep tendon reflexes in the extremities, and ataxia. Lumbar puncture 1 week after onset showed albuminocytologic dissociation and serum antibodies against GQ1b and GT1a turned out to be positive. Ultimately, the patient was diagnosed with MFS, which is a rare variant of Guillain-Barre syndrome. Because the clinical manifestations of the patient could not exclude MG, electromyography, and serum muscle weakness antibody profile were performed. The results showed positive for axillary nerve repetitive electrical stimulation and antibodies against acetylcholine receptor (AChR) and titin were detected, so the patient was diagnosed with MG at the same time. Even though only five cases of overlapping MFS and MG so far have been described, two different autoimmune diseases may coexist. When one disease presents with uncommon symptoms, careful identification of the presence or absence of other comorbid diseases should be required.

## Background

Miller Fisher syndrome (MFS) is a rare variant of Guillain-Barre syndrome (GBS), an acute, immune-mediated, monophasic disease that usually presents as ocular muscle paralysis, dysreflexia, and ataxia. The worldwide incidence of GBS is estimated to be 1–2 per 100,000 people. Miller-Fischer syndrome accounting for only a small fraction of the total and its prevalence is higher in Asia where it is estimated to account for 15–25% of GBS, compared to only 1–7% in the West (1). Autoimmune myasthenia gravis (MG) is an antibody-mediated chronic disease which is one of the most common disorders affecting neuromuscular transmission, in which alterations in neuromuscular transmission lead to skeletal muscle weakness and fatigability. The worldwide incidence is estimated to be between 0.3 per 100,000 people and 2.8 per 100,000 people (2). The incidences of both diseases are low, so the chance of overlap between the two diseases is very low, with only 5 cases reported worldwide (3–7).

## Case Report

A 58-year-old male patient had a sudden onset of dizziness with slurred speech, subsequently accompanied by numbness of upper extremities, choking and coughing with water, and ptosis of the right eye. Then, he went to the local hospital for consultation, and the brain MRI scan did not show any obvious abnormal signs. In order to determine further treatment, the patient was admitted to our hospital diagnosed with “ball palsy to be investigated.”

He previously had “kidney stone surgery” 3 years ago and was diagnosed with nephritis which improved after taking medication (unknown) in March 2021. In April 2021, he underwent “back lipoma resection.” The patient had not been previously exposed to the novel coronavirus and had not been vaccinated against it.

At the time of admission, the neurological examination showed ptosis of the right eye and limitation in abduction and adduction of both eyes, absent deep tendon reflexes in the extremities, positive at finger-to-nose test together with a slightly shallowness of nasolabial fold on the right side, weakness of cheek puffing on the right side, slight weakness of the neck extensor muscle, and positive of the right eyelid fatigue test. Bilateral frontal lines are basically symmetrical, tongue extension was center, muscle strength and muscle tone in the limbs was normal, deep and superficial sensation were normal, and there were negative pathological signs on both sides. Brain MRA, chest CT, carotid ultrasound, and vertebral artery ultrasound did not show any lesions related to symptoms. Blood tests were unremarkable except for mild hypercholesterolemia and high red blood cells in the urine. Tumor indicators, autoimmune antibodies, thyroid function, serum folate, and vitamin B12 dose were normal.

The symptoms of the patient were more consistent with the MFS triad. Electromyography, performed 6 days after the onset of the disease, showed axonal damage to peripheral sensory nerves of the extremities. The lumbar puncture performed 1 week after his onset of the disease showed that the cerebrospinal fluid was suggestive of cellular protein separation, and was positive for antibodies against GQ1b and GT1a in 12 items of the serum anti-ganglioside antibodies. Therefore, the diagnosis of Miller-Fisher syndrome was confirmed. On the seventh day after onset, relevant contraindications were excluded, and the patient was given immunoglobulin therapy at 0.4 mg/kg per day for 5 days. The symptoms of the patient improved significantly after one treatment period.

The patient showed mild facial palsy, dysphagia, and weakness of curved neck, which are not common symptoms of MFS. Combined with the fact that the muscle weakness of the patient was fatigue-related and the phenomenon of “light in the morning and heavy in the evening” and positive of the right eyelid fatigue test, MG was excluded. After admission, the chest CT revealed that no significant abnormality could be seen in the thymus gland. Neostigmine test was performed prior to Immunoglobulin treatment, but the results suggested that there was no significant improvement before and after the injection. Despite this, electromyography performed 13 days after the onset of the disease showed that when the axillary nerve was repeatedly electrically stimulated, the wave amplitude decreased by about 35.8% under low frequency electrical stimulation (3 Hz) and about 50.6% under high frequency electrical stimulation (10 Hz). The patient underwent repetitive electrical stimulation tests of the facial and axillary nerves twice within 1 week of admission, both of which were negative for the facial nerve and positive for the axillary nerve. Together with positive of antibodies against acetylcholine receptor (AChR) and titin, the diagnosis of myasthenia gravis was confirmed.

After a course of immunoglobulin injection, the ophthalmoplegia and dysarthria of the patient significantly improved compared with before. At the time of discharge, the patient still had a little numbness in double hands, no obvious slurred speech, no dizziness, and no headache. Neurological examination signs were significantly less severe than before.

## Discussion

Together with the five previously published cases of co-morbid MFS and MG, this is the second case in which both diseases were diagnosed simultaneously for the first time, with the first case being published in 2016 (4). The details of the five previous cases are shown in Table 1.

**Table 1:** Characters of the five cases of overlapping MFS and MG.

	**Age(y)**	**Gender**	**History of antecedent infection**	**Clinical presentation**	**Past history**	**MG antibodies**	**MFS antibodies**	**Treatment**	**Prognosis**
**Case 1 (** **3** **)**	40	Male	2 weeks Had a flu-like illness	Complete ocular muscle paralysis in both eyes, partial ptosis in the left eye, and loss of pupillary light reflex in the right eye. With loss of tendon reflexes, ataxia	MG 7 years	-	anti-GQ1b antibodies	3 times plasma exchange	Good
**Case 2 (** **4** **)**	69	Female	None	Bilateral ptosis with dysarthria after 1 week. Ataxia, loss of tendon reflexes.	Diagnosed with chronic kidney disease 2 years ago	Anti-AchR antibodies	Anti-GQ1b antibodies	First treated with immunoglobulin injections, no improvement in symptoms, then steroid hormone therapy with better results	Better
**Case 3 (** **5** **)**	84	Female	With upper respiratory tract infection 5 days ago	Ptosis, diplopia, dysphagia, and slurred speech, loss of all tendon reflexes	MG 6 year	Anti-AchR antibodies	Anti-GQ1b antibodies	IVIG	Better
**Case 4 (** **6** **)**	79	Male	Upper respiratory tract infection, influenza vaccination received a few weeks ago	Diplopia aggravated with nausea, vomiting, ataxia, loss of tendon reflexes	MG 8 years, mild medical chronic sensorimotor axonal polyneuropathy due to postoperative chemotherapy for colon cancer	Anti-RyR antibodies, anti-AchR antibodies	anti-GQ1b antibodi-es	5plasmapheresis	Better
**Case 5 (** **7** **)**	43	Male	None	Bilateral diplopia, bilateral hand sensory abnormalities, mostly absent tendon reflexes	MG diagnosed 15 years ago as anti-AchR antibody negative	None	Anti-GQ1b antibody	–	Good

In this case, the patient was positive for serum antibodies against GQ1b and GT1a in the 12 anti-ganglioside antibodies. Anti-GQ1b antibodies can cross-react with anti-GT1a antibodies in MFS. GT1a is specifically distributed in the lower part of the central nervous system and is associated with medullary paralysis. Therefore, cross-reactivity between anti-GQ1b and GT1a can lead to medullary paralysis (8). In addition, our patient presented with symptoms such as weakness in swallowing and choking on drinking water. The serum MG profile of our patient showed positive of antibodies against AChR and titin. Notably, anti-Titin antibody IgG is an antibody against intracellular components of rhabdomyocytes (9). It is not clear whether these four antibodies will appear to cross-react with each other, but there is a view that anti-ganglioside antibodies may cause lesions of the neuromuscular junction, leading to the development of muscle weakness symptoms similar to those of MG (10).

Although our patient had nephritis and surgery, antibiotics and anesthesia may have caused MG, but it did not manifest as muscle weakness at that time. Antibiotics and anesthetic supplies can all cause worsening of MG. The mechanisms of antibiotic-induced MG exacerbation include presynaptic interactions with voltage-gated calcium channels, calcium-sensitive receptors, and postsynaptic interactions with acetylcholine receptors (11). Many anesthetic supplies can also directly aggravate MG. Because the variety and dosage of anesthetics during surgery have a great impact on MG patients, anesthetic drugs that do not affect neuromuscular conduction and respiratory function should be selected for MG patients whenever possible (12).

Because both MG and MFS are rare autoimmune mediated diseases, The simultaneous diagnosis of these two diseases in one patient is a rare finding. We should distinguish carefully in the differential diagnosis of neurological diseases in the future and should not ignore the overlap of diseases.

## Data Availability Statement

The original contributions presented in the study are included in the article/supplementary material, further inquiries can be directed to the corresponding author/s.

## Author Contributions

NC contributed to the conception and drafting of the manuscript. HC contributed to acquisition of data. JC contributed to the drafting and revision of the manuscript. All authors contributed to the article and approved the submitted version.

## Conflict of Interest

The authors declare that the research was conducted in the absence of any commercial or financial relationships that could be construed as a potential conflict of interest.

## Publisher's Note

All claims expressed in this article are solely those of the authors and do not necessarily represent those of their affiliated organizations, or those of the publisher, the editors and the reviewers. Any product that may be evaluated in this article, or claim that may be made by its manufacturer, is not guaranteed or endorsed by the publisher.
